# Regional Anesthesia for High-Risk Patients Undergoing Total Knee Arthroplasty: A Case Report

**DOI:** 10.7759/cureus.55269

**Published:** 2024-02-29

**Authors:** Abdullah A Alturki, Ziad A Aljaafri, Halah Alshabraqi, Ibrahim Hassan, Ahmad Alturki, Ali A Alhandi

**Affiliations:** 1 Department of Orthopedics, King Abdulaziz Medical City, Ministry of National Guard Health Affairs, Riyadh, SAU; 2 College of Medicine, King Saud Bin Abdulaziz University for Health Sciences, Riyadh, SAU; 3 Department of Anesthesia, Dr. Sulaiman Al-Habib Hospital, Riyadh, SAU; 4 College of Medicine, Imam Mohammad Ibn Saud Islamic University, Riyadh, SAU

**Keywords:** peripheral nerve block, regional anesthesia, general anesthesia, knee arthroplasty, case report

## Abstract

Total knee arthroplasty (TKA) is a commonly performed surgery for individuals experiencing advanced knee osteoarthritis. Patients undergoing TKA can present with a variety of comorbidities, ranging from the absence of chronic illnesses to the presence of multiple health conditions. The complexity of these comorbidities can pose challenges in carrying out the desired procedure due to the elevated risk profile; this limits the anesthesia modalities that the physician can utilize. Careful consideration of patients' overall health status and personalized anesthesia approaches are crucial to ensure optimal outcomes in this diverse patient population. This case involves an eighty-year-old male with a history of multiple comorbidities who underwent a left TKA. The patient presented a high-risk profile during evaluation, classified as American Society of Anesthesiology (ASA) class IV, which made general and neuraxial anesthesia unfavorable due to high risks. Regional anesthesia was utilized as the sole modality of anesthesia and was successful. This demonstrates that regional anesthesia is a viable option when attending to patients with high risks associated with other anesthesia modalities.

## Introduction

Total knee arthroplasty (TKA) is a commonly performed procedure for individuals experiencing end-stage osteoarthritis, with excellent outcomes in alleviating pain and improving functionality that has been evaluated and reported over many years [1-2]. However, some patients are at an increased risk of complications based on their coexisting comorbidities, including elevated BMI and cardiovascular diseases [3].

Anesthetic and analgesic modalities utilized in TKA have advanced significantly to improve surgical outcomes and reduce potential complications. The two primary categories of anesthesia utilized are general anesthesia (GA) and neuraxial anesthesia (NA). A recent meta-analysis revealed that, compared to GA, NA was linked to shorter hospital stays and fewer complications after TKA [4]. Multiple studies have demonstrated a significant effect when regional anesthesia is combined with NA for postoperative pain control [5].

No study in the literature has reported the utilization of regional anesthesia when both NA and GA pose higher risks. This study presents a case in which the patient underwent a novel mix of regional anesthesia modalities to achieve a complete block of sensory impulses to proceed with the desired surgical procedure.

This article was previously presented as a meeting abstract at the 3rd International Conference on Arthroplasty and Orthopedic Surgery Meeting on December 4, 2023.

## Case presentation

**Figure 1:**
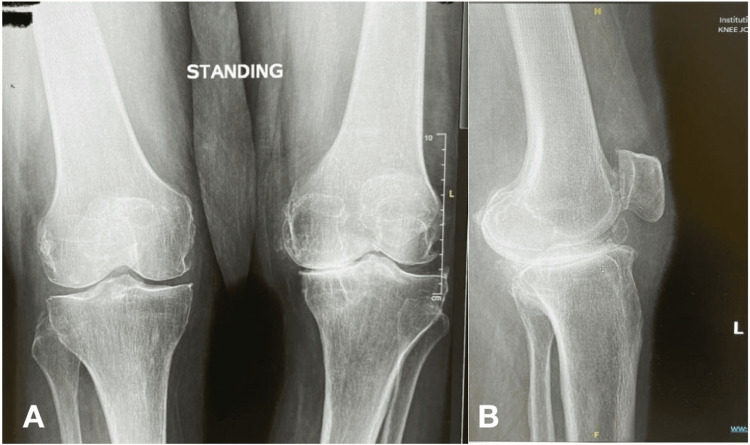
A) Anteroposterior view of the left knee showing advanced osteoarthritic changes characterized by asymmetrical joint narrowing, marginal subarticular sclerosis, and osteophyte formation. B) Lateral view of the left knee with similar changes.

An 80-year-old male with a known medical history of type 2 diabetes mellitus, benign prostatic hyperplasia, and hypertension presented to the ED, complaining of bilateral knee pain, which was worse in the left knee for the past two months. Physical examination was significant for bilateral knee swelling. Standing anteroposterior (AP) and lateral X-rays of both knees were obtained, showing advanced osteoarthritic changes in the form of asymmetrical joint narrowing, marginal subarticular sclerosis, and osteophyte formation, more so in the left knee (Figure 1). No definite fracture, dislocation, osteolytic, or sclerotic bony lesions were detected.

Upon obtaining cardiac clearance, ECG showed atrial fibrillation, while the echocardiogram showed good left ventricular ejection fraction, dilated atria, moderately severe mitral and tricuspid regurgitation, and features of restrictive cardiomyopathy were noted. Elevated pulmonary pressure (PAP) was 50 mmHg. As a result, he was noted as a moderate-risk patient from a cardiac point of view, and the anesthesiologist deemed the patient high risk for neuraxial and general anesthesia with ASA class IV.

Surgical course

The patient underwent left TKA under a regional block only with minimal sedation; no spinal or general anesthesia was used. Basic monitoring for oxygenation, ventilation, circulation, and temperature was employed, in addition to bispectral index (BIS) monitoring for depth of sedation. Midazolam 3 mg was given, then a dexmedetomidine infusion at 0.2 mcg/kg/hr. Under ultrasound guidance, the patient was placed in the lateral position, and an infra-gluteal para-biceps approach was used for sciatic nerve blockade (SNB), with 15 mL of 0.75% Ropivacaine with 1:200,000 epinephrine given deep to the common investing extra-neural layer around the sciatic nerve. With the patient supine, the lateral femoral cutaneous nerve (LFCN) was identified lateral to the sartorius muscle, anterior to the iliotibial tract, and below the fascia lata. Five mL of 0.75% ropivacaine with 1:200,000 epinephrine was injected in this area. With the patient supine and legs in a figure-of-four position, the distal divisions of the obturator nerve were identified between the adductor longus and brevis muscles (anterior division) and between the adductor brevis and magnus (posterior division). A total of 10 mL of 0.75% ropivacaine with 1:200,000 epinephrine was used for the obturator nerve block, 5 mL for each division. Finally, with the patient supine, the femoral nerve was located below the fascia iliaca and lateral to the femoral artery. 15 mL of 0.75% Ropivacaine with 1:200,000 epinephrine was administered deep to the fascia iliaca, surrounding the nerve [6].

The patient was placed in a supine position; a tourniquet was applied but not used during the surgery. Draping was performed in the usual sterile manner. A medial parapatellar approach was utilized. The implant used for the patient’s total knee was the Genesis II PS Oxinium from Smith & Nephew, with both components, femoral and tibial, cemented and inserted in the usual manner. Good hemostasis was achieved during the surgery, and the range of motion was tested with good patellar tracking and stability with varus and valgus stress. Finally, closure of the tendon was done in the usual fashion using Vicryl and Stratafix, and closure in layers was done afterward.

Post-operative analgesia

An adductor canal catheter was placed in a sterile manner. A 10 cm 18-gauge stimulating Tuohy needle, connected to the negative lead of a constant voltage nerve stimulator (Stimuplex DIG; B-Braun/McGaw Medical, Bethlehem, PA), was inserted using ultrasound guidance just anterior to the femoral artery, deep to the sartorius muscle to deposit 10 mL of Ropivacaine 0.2% until its spread around the artery was confirmed with ultrasound visualization. With a vastus medialis evoked motor response (EMR) at 0.5 mA, a 20 G stimulating catheter was used while maintaining the evoked motor response of the vastus medialis muscle throughout catheter advancement. The femoral catheter was advanced 5 cm beyond the needle tip. Then, the catheter was fixed and connected to a BAXTER elastomeric pump containing 0.2% Ropivacaine with a continuous infusion of 7 mL/hr.

**Figure 2:**
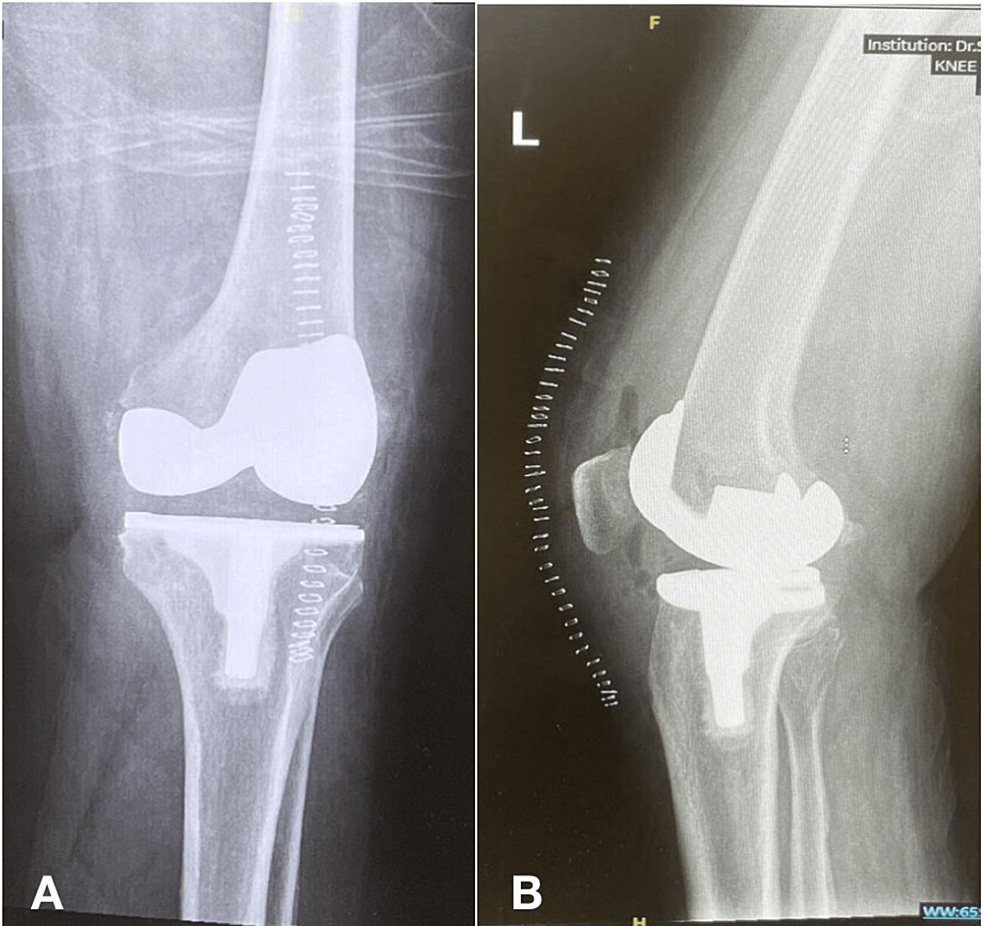
A) Anteroposterior and B) Lateral views of the left knee post-total knee replacement, showing the implant in good position.

The patient tolerated the procedure well and was transferred to the post-anesthesia care unit, then later shifted to the ward in a good, stable condition, and pain was well controlled. Lateral and AP X-rays of the left knee were obtained after the procedure, showing the implants to be in a good position (Figure 2).

## Discussion

TKA can be performed under general, neuraxial, or combination anesthesia, depending on the patient's health status and ability to tolerate the anesthesia's side effects. In scenarios where GA or NA poses a high risk, an alternative regional anesthesia technique can be utilized. Peripheral nerve block (PNB) of major nerves supplying the lower limbs can provide effective unilateral analgesia with a decreased incidence of opioid-related autonomic side effects, less motor blockade, and neurological complications. TKA becomes a safer option with more efficient outcomes, minimal adverse effects, and improved pain tolerance if PNB is continued until discharge [7-9]. In our case, TKA under regional anesthesia was successful, with a hospital stay without any complications and good outcomes regarding the prosthesis.

This case highlights the potential benefits of using regional anesthesia as an alternative to other modalities. However, using peripheral nerve blocks alone to perform TKA may have several limitations and considerations. The total dose of local anesthetic used in this case approaches the maximum recommended dose, and the technique requires multiple separate needle entry points and perineural injections. No studies have evaluated the incidence of neurologic sequelae after performing femoral, sciatic, and multiple cutaneous PNBs, similar to the technique used in this case. The innervation of the knee is highly variable, making complete surgical anesthesia challenging using PNBs alone. The methods described may aid other anesthesiologists when facing patients with high risks of GA or NA.

## Conclusions

With excellent anesthesia care, such complex cases can be managed through a patient-tailored approach. The use of PNB could be considered as the main modality for performing TKA in cases where the use of GA or NA poses a higher risk. It's important to consider the limitations of the total dose used and the advantages of continuing the PNB for post-operative pain control. Due to the patient's medical history, which includes multiple comorbidities, TKA was performed under PNB, resulting in successful outcomes with no complications.
